# Relationship between total and segmental bone mineral density and different domains of physical activity among children and adolescents: cross-sectional study

**DOI:** 10.1590/1516-3180.2017.0042070417

**Published:** 2017-08-21

**Authors:** Tiego Aparecido Diniz, Ricardo Ribeiro Agostinete, Paulo Costa, Bruna Thamyres Ciccotti Saraiva, Diego Kanashiro Sonvenso, Ismael Forte Freitas, Rômulo Araujo Fernandes, Diego Giulliano Destro Christofaro

**Affiliations:** I Doctoral Student, Department of Cell and Developmental Biology, Institute of Biomedical Sciences, University of São Paulo, São Paulo (SP), Brazil.; II Master’s Student, Department of Physiotherapy, Universidade Estadual Paulista Júlio de Mesquita Filho (UNESP), Presidente Prudente (SP), Brazil.; III Master’s Student, Department of Physical Exercise, Universidade Estadual Paulista Júlio de Mesquita Filho (UNESP), São Paulo (SP), Brazil.; IV Undergraduate Student, Department of Physical Exercise, Universidade Estadual Paulista Júlio de Mesquita Filho (UNESP), São Paulo (SP), Brazil.; V Associate Professor, Department of Physical Exercise, Department of Physical Education, Universidade Estadual Paulista Júlio de Mesquita Filho (UNESP), Presidente Prudente (SP), Brazil.

**Keywords:** Bone density, Leisure activities, Motor activity, Sports

## Abstract

**BACKGROUND:**

This study aimed to investigate the relationship between total and segmental bone mineral density (BDM) and physical activity (PA) in different domains (school, leisure and sports) among adolescents and children.

**DESIGN AND SETTING:**

Cross-sectional study in the Universidade Estadual Paulista Júlio de Mesquita Filho (UNESP).

**METHODS:**

The study sample consisted of 173 children and adolescents (10.31 ± 1.87 years). The BMDs for the whole body (WB) and the regions of the trunk and legs were measured using dual energy X-ray absorptiometry (DXA). PA was measured using the Baecke questionnaire. A regression model was used to analyze the relationship between all the BMDs and the different domains of PA.

**RESULTS:**

41.5% of the adolescents had high percentages of body fat. Regarding the comparison between physically active and insufficiently active adolescents, there were no statistically significant differences in any BMD variables (P > 0.05). The BMD of the legs showed positive relationships with the total PA (β = 0.009; P = 0.013) and sports PA (β = 0.010; P = 0.049) after insertion of the confounders. Similarly, the WB BMD showed the same relationships (total PA: β = 0.005; P = 0.045; and sports PA: β = 0.008; P = 0.049). No relationship was found between leisure and school PA and any of the BMDs (P > 0.05).

**CONCLUSION:**

The results indicated that practice of sport was related to higher BMD values, independent of sex, age and body fatness.

## INTRODUCTION

Development of human tissue, including bone tissue, is determined by biological events during childhood and adolescence.^1^ Bone mineral density (BMD) represents the amount of inorganic material (calcium and phosphorus) stored in the bones, which varies over the course of life. It can be measured either for the whole body or in segments.^1^^,^^2^ Low BMD values are related to development of osteoporosis, mainly in later life, but also in pediatric populations.^2^ Bone health in adulthood is determined by bone development over the course of early life, which can be affected by a large variety of variables, such as genetics, nutrition, hormone action, biological maturation and physical activity (PA).^3^^,^^4^

PA exerts significant influence on BMD accrual during growth,^4^ which can be categorized into domains (school, leisure and sports). School PA denotes activities performed during school activities,^5^^,^^6^ while leisure PA denotes activities performed during free time.^7^^,^^8^^,^^9^ Moreover, in young populations, sport PA can itself be considered to be a PA domain.^10^^,^^11^^,^^12^

PA at vigorous intensity, including mechanical loading on the bones, positively affects bone mass due to:

1. Muscle action, which promotes high load and stress on the bones, thereby affecting and modifying bone strength and geometry;
2. The rate of bone turnover, which is modulated by the action of osteoblast (formation) and osteoclast (resorption) systems, which in turn promote significant gains in BMD.^13^

In this way, PA can have a greater influence on some specific BMD segments in the body.^3^^,^^11^ The BMD of the legs comprises one component of weight-bearing joints and may be indicative of a specific site where bone-loading occurs and tends to have greater impact. On the other hand, upper limbs are used more specifically in activities such as combat sports^11^ and gymnastics.^14^ Thus, it appears to be of interest to investigate the effect of PA on BMD in different body segments.

Moreover, although BMD and PA have been correlated in studies involving organized physical activity (physical exercise and types of sport),^15^ there are fewer data on the relationship between bone health and leisure PA. The absence of data on this issue is more relevant among young people, because important confounders (body fat, age and gender) affect growth and it is not clear whether the impact of PA is independent of these confounders.

## OBJECTIVE

Thus, the objectives of this study were to compare BDM (both total and segmental), between physically active and insufficiently active adolescents, and to evaluate the relationship between the practice of physical activities in different domains (school, sports and leisure), and whole-body and appendicular BMD.

## METHODS

### Compliance with ethical standards

This study was approved by the ethics board of Universidade Estadual Paulista Júlio de Mesquita Filho (UNESP), Presidente Prudente campus (procedural number: 26702414.0.0000.5402). All procedures performed in this study were in accordance with the ethical standards of the institutional and/or the national research committee, and with the 1964 Helsinki declaration and its later amendments or comparable ethical standards. Written consent was obtained from all parents before the adolescents were included in the study.

### Study design and subjects

This was a cross-sectional study evaluating the level of physical activity and BDM among adolescents in Presidente Prudente at Universidade Estadual Paulista Júlio de Mesquita Filho (UNESP).

The study sample was composed of 173 adolescents, aged between 10-14 years (mean = 11.68 years; standard deviation, SD = 1.44), who formed part of a Brazilian social project with activities at a philanthropic institution in Presidente Prudente, state of São Paulo, Brazil. All the children and adolescents of this social project were invited to participate in the study and those who accepted formed part of the study sample. All participants presented a consent statement signed by a parent or guardian, authorizing them to participate in this study.

### Anthropometric measurements and biological maturation

Body mass was obtained using a digital scale accurate to 0.1 kg. Height was measured using a fixed stadiometer accurate to 0.1 cm, with a maximum length of two meters. From these measurements, body mass index (BMI) was calculated and the z score was generated. Leg length and seated height were measured using standardized techniques. These measurements were used to calculate the maturity offset, which denotes the time (years) from/to the age of peak height velocity (APHV), which is an important maturational event.^16^

### Bone mineral density

Dual energy X-ray absorptiometry (DXA) was used to assess BDM. DXA can be used to analyze the whole-body, trunk region and leg BMDs (WB BDM, trunk BMD and leg BDM, respectively) in g/m^2^, along with the percentage body fat (%BF). The equipment used was the Lunar-DPX-NT model (General Electric, GE). The results were expressed as means, which were calculated using specific software supplied with the equipment.

Body fat levels were classified high if they were above 25% and above 30% for male and female adolescents respectively, in accordance with the cutoffs proposed by Williams et al.^17^

### Physical activity

Habitual PA was assessed using the questionnaire developed by Baecke et al.,^10^ which has been validated for use among Brazilian adolescents.^18^ This questionnaire assesses habitual PA according to three different domains: school, leisure and sports outside school. The school score was assessed from questions relating to the amount of time spent performing the following activities during school time: sitting, standing, walking, lifting heavy loads, fatigue and sweating. The leisure PA score specifically asked about time spent on watching television, walking, cycling and active transportation (via walking and/or cycling). Finally, the sport PA score was calculated by asking about which two specific sports the adolescent participated in most frequently, their number of hours per week and months per year of participation and their degree of sweating; and by making comparisons of PA levels with other individuals of the same age. The total score was calculated by adding together the individual scores for school, leisure and sport PA.

Moreover, this questionnaire had previously been validated against the gold standard method (doubly labeled water) for measurement of PA.^19^ Children and adolescents located in the highest quartile of total PA (Q4) were considered to be physically active, while children and adolescents in the intermediate quartiles (Q2 and Q3) were classified as moderately active and those in the lowest quartile (Q1) were classified as inactive.

### Statistical analysis

The data were subjected to the Kolmogorov-Smirnov test to verify normality. If the distribution was found to be normal, the variables of the sample were characterized in terms of the mean and standard deviation. The Pearson correlation was used to examine the relationship between PA and BMD values among the adolescents. Regression models were used to evaluate the relationship between BMD and PA (treated in this statistical analysis in the continuous form) either with each PA domain or with the total PA, using independent variables. In the first multivariate analysis, the variables of sex and age were inserted in order to eliminate possible confounding factors when analyzing the relationships between the different PA domains and trunk BMD, leg BMD and WB BMD. In the second multivariate model, %BF assessed by means of DXA and maturation was inserted to verify whether the relationships between the PA domains and trunk BMD, leg BMD and WB BMD remained. Firstly, the relationship between the different domains of physical activity and bone mineral density was evaluated separately; and secondly, these domains were inserted simultaneously in order to verify whether any of the domains overlapped on the others (e.g. situations in which physical activity in school and sports practice were correlated with higher bone mineral density of the legs, both when analyzed separately and when inserted simultaneously). The statistical significance level adopted was 5%.

## RESULTS

The DXA evaluation showed that the percentage of the adolescents with high levels of body fat (≥ 25% for boys and ≥ 30% for girls) was 41.5%. Girls had a higher percentage of body fat than boys: 38.0% and 49.5% respectively. Table 1 shows the characteristic information of the sample according to the PA level (physically active, moderately or inactive).

**Table 1.**
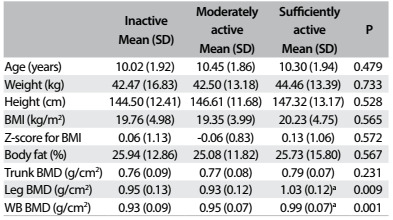
Characteristics of the subjects according to physical active level

SD = standard deviation; BMI = body mass index; BMD = bone mineral density; WB = whole body. ^a^statistically significant in relation to the inactive group.

The following PA correlations among the young people were not significant: between PA at school and trunk BMD (r = 0.14; P = 0.063); between PA in sports activities outside the school environment and trunk BMD (r = 0.13; P = 0.077); and between PA during leisure time and trunk BMD (r = 0.06; P = 0.430). Table 2 shows the multivariate analysis information on the relationship between BMD and the different physical activity domains. No significant relationships between the different domains of physical activity and trunk BMD were observed.

**Table 2.**
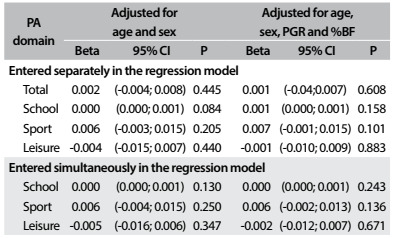
Relationship between trunk bone mineral density and different physical activity domains

%BF = body fat: PA = physical activity; PGR = peak growth rate; 95% CI = 95% confidence interval; SD = standard deviation.

PA performed by the young people at school was not significantly related to leg BMD (r = 0.13; P = 0.075). However, PA during leisure time showed a significant relationship with leg BMD (r = 0.21; P = 0.005) and practicing sports activities outside the school was significantly associated with leg BMD (r = 0.20; P = 0.008). In Table 3, in the multivariate analysis, only the sports practice and total PA were related to higher leg BMD.

**Table 3.**
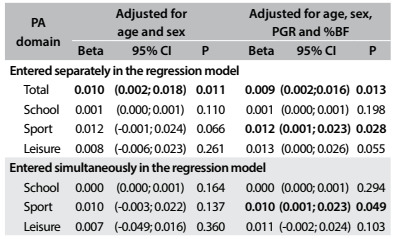
Relationship between leg bone mineral density and different physical activity domains

%BF = body fat: PA = physical activity; PGR = peak growth rate; 95% CI = 95% confidence interval; SD = standard deviation.

PA at school was not significantly related to WB BMD (r = 0.16; P = 0.111). PA in sports was related to WB BMD (r = 0.18; P = 0.003) and PA during leisure time did not show any significant relationship with WB BMD (r = -0.13; P = 0.079). Sports practice and total PA were correlated with higher WB BMD in the multivariate analysis. This information is shown in Table 4.

**Table 4.**
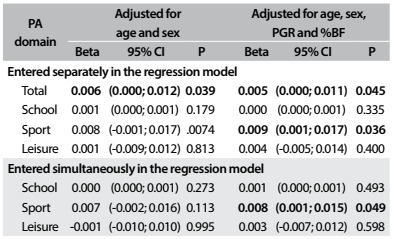
Relationship between whole body bone mineral density and different physical activity domains

%BF = body fat: PA = physical activity; PGR = peak growth rate; 95% CI = 95% confidence interval; SD = standard deviation.

The sum of the three PA domains was unrelated to trunk BMD (r = 0.06; P = 0.418), but this sum had statistically significant relationships with leg BMD (r = 0.17; P = 0.027) and WB BMD (r = 0.15; P = 0.048).

After all adjustments, with the variables entered separately in the multivariate models, it was observed that PA at school and during leisure time was not related to the different BMDs analyzed (P > 0.05). Sports practice among the adolescents was significantly related to higher leg BMD (β = 0.012; 95% confidence interval, CI = 0.001-0.023; P = 0.028) and WB BMD (β = 0.009; 95% CI = 0.001-0.017; P = 0.036) (Tables 2, 3 and 4). Regarding the total PA, which was the sum of the three PA domains, we found positive relationships with leg BMD (β = 0.009; 95% CI = 0.002-0.016; P = 0.013) and WB BMD (β = 0.005; 95% CI = 0.000-0.011; P = 0.045). When the variables were entered simultaneously in the regression model, only the relationships between the sports domain and leg BMD (β = 0.010; 95% CI = 0.001-0.023; P = 0.049) and WB BMD (β = 0.008; 95% CI = 0.001- 0.015; P = 0.049) remained significant.

## DISCUSSION

The relationship between PA in different domains (school, sports and leisure) and whole-body and segmental BMD measured by means of DXA among children and adolescents was examined. After adjusting for confounding variables (age, sex and %BF), the analysis showed that sports practice had a positive relationship with total and segmental BMD, and with total PA analyzed as the sum of the three different PA domains.

PA practice appears to be essential for maintaining bone health.^20^ However, studies have demonstrated that only physical activities of moderate and vigorous intensity benefit BMD.^5^^,^^13^ In this respect, our study showed that the sufficiently active adolescents did not show higher total and segmented BMD than that of the insufficiently active (Table 1). Corroborating our findings, Gracia-Marco et al.^21^ found that children classified as physically active showed no differences in BMD, compared with sedentary individuals. Their categorization took total PA into account, which may have included sedentary and light activities, which have a less positive relationship regarding addition of BMD.^22^

The present study demonstrated that total PA (i.e. the sum of school, sports and leisure PA) presented a positive relationship with WB BMD, trunk BMD and leg BMD, even after all statistical adjustments. Corroborating this, Tobias et al.^22^ showed that practicing moderate to vigorous PA had a positive correlation with the BMD of the lower limbs after adjustments for height, lean body mass and body fat. Neville et al.^23^ found data similar to ours, i.e. that the sum of PA, derived from the Baecke questionnaire, was positively associated with increases in lumbar spine and femoral neck BMD. However, the participants in the sample of Neville et al.^23^ were 15 years or over, while it has been reported that the greatest accrual of bone mass occurs at around 13 and 11 years, respectively, in boys and girls.^11^ Furthermore, the Baecke questionnaire was used only to evaluate the total PA, thus missing a lot of information regarding the different PA domains (school, sports and leisure).

Therefore, we investigated the relationships of all PA domains with total and segmental BMD. It was found that physical activity at school showed no relationship with total or segmental BMD. Agreeing with our results, Heidemann et al.^5^ found from a two-year follow-up that increased physical activity at school (e.g. the number of days of physical education) did not give rise to any significant increase in BMD. Valdimarsson et al.^6^ found that girls who engaged in more than 3 hours of physical education at school per week during a one-year follow-up did not present higher total and leg BMD than those at a traditional school (1 hour of physical education per week). These findings suggest that physical activity performed only in a school environment is insufficient to generate increases in BMD. Since no details on the type of activities performed in these interventions were reported in those studies, we assume that these findings were due to low weight-bearing activities in the interventions.

On the other hand, some studies have demonstrated beneficial effects on BMD sites from interventions during school time, even after three years.^24^ In the study by Meyer et al.,^24^ the physical education classes were composed of a multi-component PA intervention that included daily physical education with at least 10 minutes of jumping or strength-training exercises of various intensities. A similar protocol was used by Heidemann et al.,^5^ comprising increased numbers of physical education classes, but the results were different. In their study, the adolescents who participated in the nine-month intervention program demonstrated increased total BMD. However, comparisons between the results from these studies showed that there were some limitations regarding the type, frequency and duration of PA and pubertal maturation, which makes it difficult to establish a pattern in the relationship between school PA and BMD.

In our investigation on the relationship between leisure PA and BMD sites, it was found that school PA was unlikely to be sufficient to increase total and segmental BMD. In the Baecke questionnaire, there are two important questions in this section that address how long adolescents spend walking and/or cycling. Thus, adolescents who are active with regard to leisure PA must spend more time on these activities. Park et al.^9^ found that the practice of regular walking was not positively correlated with total or segmental BMD in adolescents. Corroborating this, in the study by Deere et al.^7^ adolescents who practiced running or high-impact activities presented higher values for hip BMD. In contrast, the practice of jogging showed little benefit at the BMD sites. Another type of leisure PA considered in the questionnaire was cycling. A large number of studies have demonstrated that the practice of cycling is insufficient to increase total and segmental BMD.^8^^,^^15^ These results demonstrated that physical activity performed without or with little weight-bearing showed no benefits at the BMD sites.

Moreover, in our study, the practice of sports PA was related to whole-body and leg BMD, even after introduction of confounding variables in the multivariate model. Corroborating this, Nasri et al.^11^ found that adolescents who practiced combat sports had higher values for total hip and lumbar spine (L2-L4) BMD, compared with sedentary individuals. Silva et al.^12^ found that adolescents who engaged in practicing sports such as soccer and tennis had greater BMD than the control group. On the other hand, adolescents who practiced swimming did not present increased BMD.

Furthermore, sports can be categorized as vigorous PA. It is known that vigorous PA promotes gains in BMD.^11^ In this regard, Cardadeiro et al.^13^ found that an additional 10 minutes of vigorous PA per day suggested a 1-2% increase in BMD in children. These findings are consistent with the results from a 15-year monitoring epidemiological study.^25^ Moreover, Heidemann et al.^5^ found similar findings in their two-year follow-up. Adolescents who increased their amounts of high-intensity PA had greater gains in BMD than did those with lower levels.

These positive relationships between PA, especially sports, and total and segmental BMD can be explained by the action of osteocytes, which are embedded within the mineralized bone. In response to mechanical loads or microlesions, these provide signals to osteoclasts, which carry out resorption. Moreover, it is known that in pre-pubertal children, the osteogenic process is more sensitive to the mechanical load in the bone, and this can augment the duration of the peak bone mass.^4^^,^^16^ Taken together, these data suggest that performing sport during adolescence shows great benefits for bone mass, and thus, may prevent development of early osteoporosis.^26^

Despite the importance of the results found here, it is important to mention some limitations. The cross-sectional design does not allow any consideration of the effect of time on these adolescents and thus does not allow causal inferences. Some of the results were borderline, meaning that the sample size was probably small. Measuring PA by means of the questionnaire of Baecke et al.^10^ may involve self-reporting errors, since it depends on the reviewers’ perception. However, among the questionnaires commonly used in epidemiological studies, use of the one described by Baecke et al.^10^ seems to be a good strategy for mitigating the limitations inherent in questionnaires, since it has a high correlation with the gold standard for estimating PA. Furthermore, use of this questionnaire allowed us to analyze the different domains of PA, thereby indicating how to increase BMD and where public policy should act to provide the means for practicing PA.

## CONCLUSION

In summary, in this sample composed of adolescents, sport practice was correlated with higher BMD values, independent of sex, age and body fatness.
